# Prevalence of ‘pouch failure’ of the ileoanal pouch in ulcerative colitis: a systematic review and meta-analysis

**DOI:** 10.1007/s00384-021-04067-6

**Published:** 2021-11-26

**Authors:** Zaid Alsafi, Alice Snell, Jonathan P. Segal

**Affiliations:** 1 Imperial College London, London, UK; 2 Surrey and Sussex Healthcare NHS Trust, Redhill, UK; 3 Department of Gastroenterology, Imperial College Healthcare NHS Trust, London, UK

**Keywords:** Ulcerative colitis, Ileal pouch-anal anastomosis, Pouch failure

## Abstract

**Background and aims:**

The ileoanal pouch (IPAA) provides patients with ulcerative colitis (UC) that have not responded to medical therapy an option to retain bowel continuity and defecate without the need for a long-term stoma. Despite good functional outcomes, some pouches fail, requiring permanent diversion, pouchectomy, or a redo pouch. The incidence of pouch failure ranges between 2 and 15% in the literature. We conducted a systematic review and meta-analysis aiming to define the prevalence of pouch failure in patients with UC who have undergone IPAA using population-based studies.

**Methods:**

We searched Embase, Embase classic and PubMed from 1978 to 31st of May 2021 to identify cross-sectional studies that reported the prevalence of pouch failure in adults (≥ 18 years of age) who underwent IPAA for UC.

**Results:**

Twenty-six studies comprising 23,389 patients were analysed. With < 5 years of follow-up, the prevalence of pouch failure was 5% (95%CI 3–10%). With ≥ 5 but < 10 years of follow-up, the prevalence was 5% (95%CI 4–7%). This increased to 9% (95%CI 7–16%) with ≥ 10 years of follow-up. The overall prevalence of pouch failure was 6% (95%CI 5–8%).

**Conclusions:**

The overall prevalence of pouch failure in patients over the age of 18 who have undergone restorative proctocolectomy in UC is 6%. These data are important for counselling patients considering this operation. Importantly, for those patients with UC being considered for a pouch, their disease course has often resulted in both physical and psychological morbidity and hence providing accurate expectations for these patients is vital.

**Supplementary information:**

The online version contains supplementary material available at 10.1007/s00384-021-04067-6.

## Introduction

Restorative proctocolectomy with ileal pouch-anal anastomosis (IPAA) is considered to be the gold standard surgical treatment for ulcerative colitis (UC) that is refractory to medical therapy [1]. Several iterations have been described in the literature following its introduction in 1978 by Parks and Nicholls [2]. Ultimately, IPAA involves resection of the diseased colon and rectum and restoration of bowel continuity, allowing the patient to defecate without the need of an ileostomy. This in turn has been shown to improve patients’ quality of life [3]. The value of such a procedure cannot be understated when considering that one fifth of patients with UC will need surgical intervention with a colectomy rate of 16% after a disease duration of 10 years [4].

Despite good functional outcomes, serious complications such as pelvic sepsis, strictures, anastomotic leaks, de novo Crohn’s disease, pouchitis and persistent pouch dysfunction can occur. These are known risk factors for pouch failure, defined as the need for pouch resection, permanent diversion, or a redo pouch. Several risk factors for pouch failure have been documented in the literature, the commonest being pelvic sepsis [5]. Chronic pouchitis and fistulas have also been associated with pouch failure. Primary sclerosing cholangitis can increase pouchitis rates as well as the risk of postoperative sepsis [6]. Reported failure rates vary significantly in the literature, ranging from 2 to 15% [7, 8]. When compared to a permanent ileostomy, a viable alternative, IPAA has been shown to improve patients’ perceptions of their body image and has similar effects on quality of life [9]. Despite this, IPAA has a higher complication rate [10]. A vital component of informed decision making and managing expectations is being able to accurately convey the likelihood of complications such as pouch failure occurring for a given intervention. Furthermore, understanding the prevalence of pouch failure and how this changes over time can help identify and manage complications which have the potential to cause pouch failure.

Therefore, we conducted a systematic review and meta-analysis aiming to define the prevalence of pouch failure in patients with UC exclusively who have undergone IPAA using population-based studies.

## Methods

We searched Embase, Embase classic and PubMed from 1978 to 31st of May 2021 to identify population-based studies (cross-sectional, case control and cohort) that reported the prevalence of pouch failure in adults (≥ 90% of the population), age 18 years and above who underwent IPAA for UC exclusively. Pouch failure was defined as the need for permanent diversion and/or pouchectomy and/or a redo pouch. Short-term outcomes have been reported extensively in the literature [11]. Therefore, we only included studies with a minimum of 1-year follow-up. To minimise the risk of selection bias, we excluded studies with a small sample size (< 200 patients). We hand-searched the references from eligible studies and the proceedings from inflammatory bowel disease conferences (United European Gastroenterology, European Crohn’s and Colitis Society, British Society of Gastroenterology and Digestive Disease week) up until May 2021. We searched the medical literature using the terms provided in Supplementary Table 1, using them as medical subject headings [MeSH] and free-text terms. No language restrictions were implemented. We manually searched references from eligible studies for any further studies to included. For studies that appeared to be eligible for inclusion but did not have sufficient data, we attempted to contact the authors for clarification.

Data were extracted by two independent reviewers (ZA, AS) using Microsoft Excel spreadsheets. The extracted data is provided in Supplementary Table 2. All disagreements went to a third reviewer (JPS) for a consensus. The following data were extracted for each study: country, number of patients providing complete data, type of study, year of study, reason for pouch failure, total number of patients, number of patients with pouch failure, mean/median follow-up (rounded to the nearest year), number of failed IPAA at various timepoints.

We combined the proportion of patients with pouch failure in each study to give a pooled prevalence for each study. We then performed a random effects model to pool the data and provide an estimate of the prevalence of pouch failure.

We assessed the quality of case–control studies using the Newcastle–Ottawa scale, with a total possible score of 9 (higher scores indicating higher quality studies). Sensitivity analysis was performed using the one-study remove method to detect the impact of each study on the combined effect. Publication bias was assessed by funnel plot inspection and Egger’s test. Heterogeneity was assessed using the *I*^2^ statistic. All statistics were performed using R with the package “meta.”

This review is presented in line with the PRISMA guidelines and was registered priori on Prospero (CRD42021259505).

## Results

**Fig. 1:**
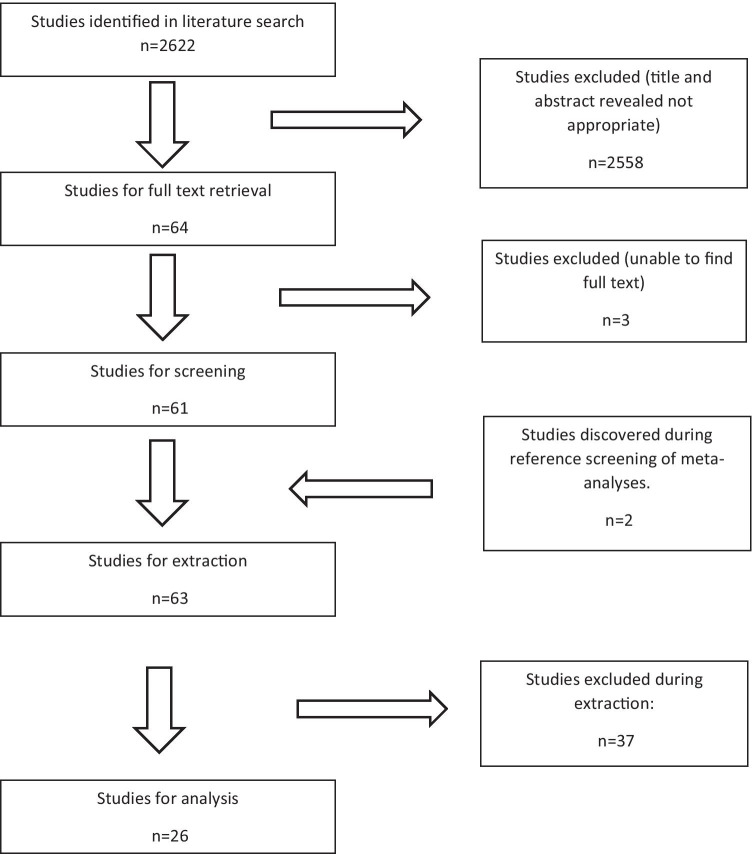
PRISMA flowchart

Two thousand six hundred and twenty-two studies were identified in the search. Twenty-six cohort studies (5 prospective cohort studies and 21 retrospective cohort studies) met the inclusion criteria and were included in the meta-analysis [1, 5, 7, 8, 12–33]. Data were extracted from the 26 articles comprising 23,389 patients with UC who underwent IPAA (Fig. 1).

**Fig. 2:**
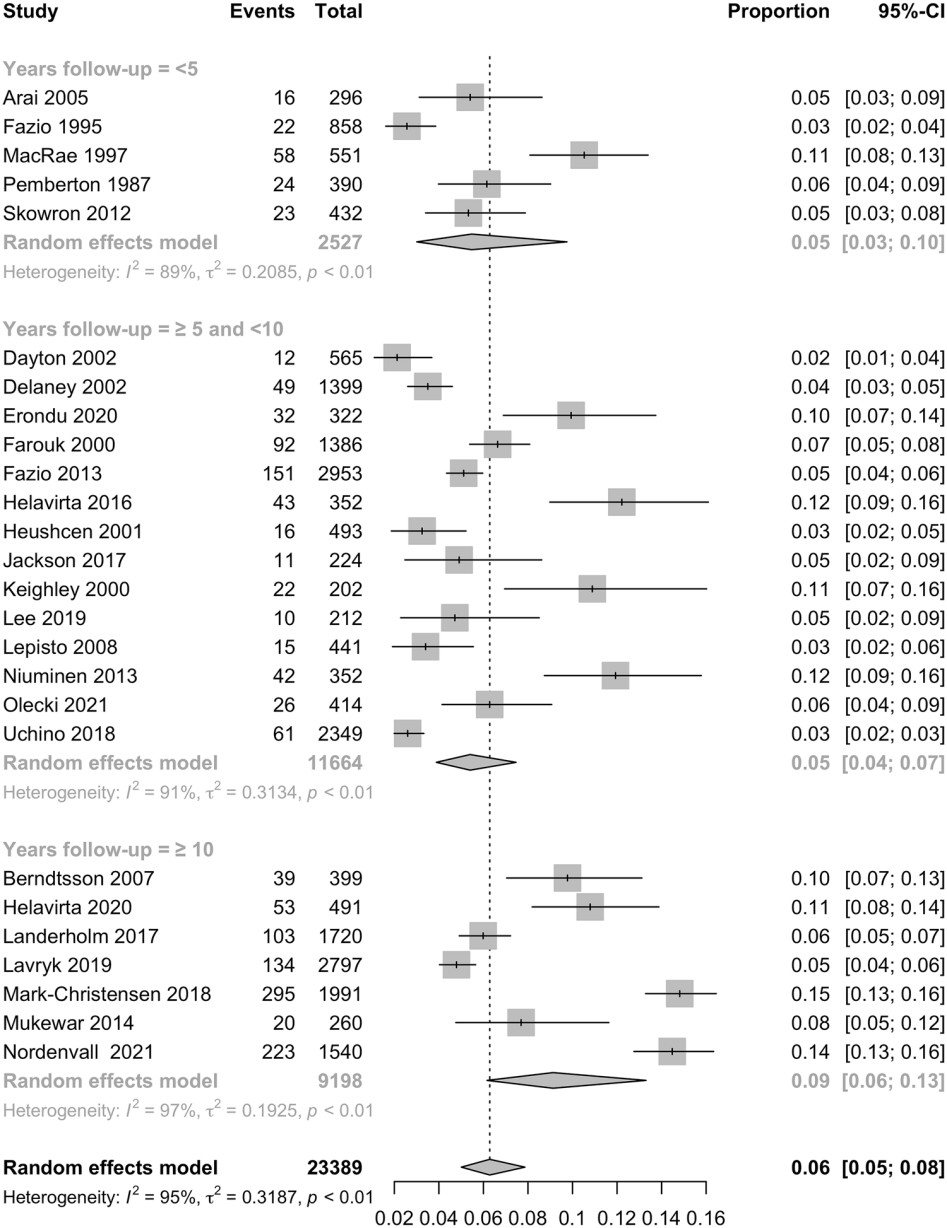
Prevalence of pouch failure

**Fig. 3:**
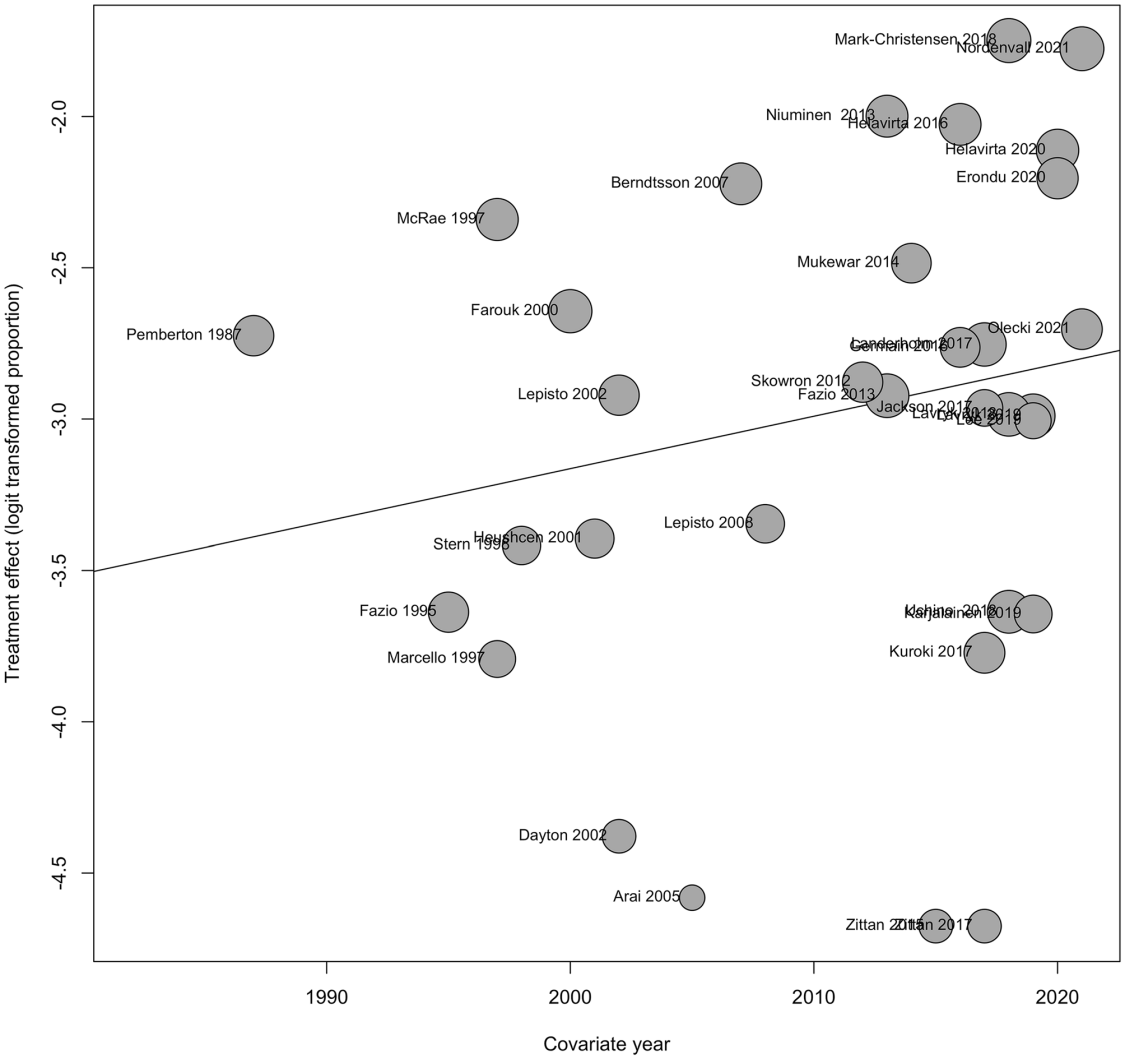
Bubble plot exploring the impact of publication year on pouch failure rate

With < 5 years of follow-up, the prevalence of pouch failure was 5% (95%CI 3–10%). With ≥ 5 but < 10 years of follow-up, the prevalence was 5% (95%CI 4–7%). This increased to 9% (95%CI 7–16%) with ≥ 10 years of follow-up. The overall prevalence of pouch failure was 6% (95%CI 5–8%) (Fig. 2). However, the studies demonstrated a significant amount of heterogeneity at each time frame (< 5 years (*I*^2^ = 89%, *P* < 0.01), ≥ 5 but < 10 (*I*^2^ = 91%, *P* < 0.01), ≥ 10 years (*I*^2^ = 97%, *P* < 0.01) and overall prevalence of pouch failure (*I*^2^ = 95%, *P* < 0.01)). Using Egger’s test, no funnel plot asymmetry was observed when assessing studies with < 5 years and ≥ 5 but < 10 years of follow-up (Supplementary Figs. 1 and 2). Due to the lack of data, it was not possible to perform Egger’s test on studies with ≥ 10 years of follow-up. Despite this, no asymmetry is observed on visual inspection of the graph (Supplementary Fig. 3). The year in which a study was published (1978–2021) did not have a significant impact on pouch failure rates (*R*^2^ = 5.12%, *P* = 0.2477) (Fig. 3).

Using the Newcastle–Ottawa scale and AHRQ standards, 19 studies were classified as poor while 7 were deemed to be good (Table 1).

**Table 1:** Newcastle–Ottawa scale and AHRQ standards assessment of studies

Newcastle–Ottawa scale and AHRQ standards assessment of studies				
Studies	Newcastle–Ottawa scale			AHRQ standards
	Selection score	Comparability score	Outcome score	
Olecki et al. [12]	3	0	2	Poor
Erondu et al. [13]	3	0	2	Poor
Lavryk et al. [14]	3	1	3	Good
Lee et al. [15]	3	1	2	Good
Jackson et al. [1]	3	0	3	Poor
Skowron et al. [16]	3	1	2	Good
Lepistö et al. [17]	3	0	2	Poor
Mark-Christensen et al. [8]	3	2	2	Good
Nordenvall et al. [18]	3	2	3	Good
Uchino et al. [5]	3	0	3	Poor
Helavirta et al. [19]	3	0	3	Poor
Landerholm et al. [20]	3	1	2	Good
Helavirta et al. [21]	3	0	2	Poor
Mukewar et al. [22]	3	1	3	Good
Nieminen et al. [23]	3	0	2	Poor
Keighley et al. [24]	2	0	1	Poor
Delaney et al. [25]	3	0	2	Poor
Dayton et al. [7]	3	0	2	Poor
Heushcen et al. [26]	3	0	2	Poor
Fazio et al. [27]	3	0	2	Poor
Berndtsson et al. [28]	2	0	2	Poor
Arai et al. [29]	2	0	1	Poor
Farouk et al. [30]	3	0	2	Poor
Fazio et al. [31]	3	0	2	Poor
Pemberton et al. [32]	2	0	2	Poor
MacRae et al. [33]	3	0	3	Poor

Nine articles reported causes of pouch failure, the most common being fistulae (*n* = 72). Other reported causes of pouch failure include pouchitis (*n* = 33), strictures (*n* = 20), Crohn’s disease (*n* = 45), surgical complications (*n* = 6) and pelvis sepsis (*n* = 1). Four articles reported failure rates at multiple time points (Supplementary Table 2).

## Discussion

To the best of our knowledge, this is the first meta-analysis with systematic review that defines the prevalence of pouch failure in patients exclusively with UC. Our data suggests that the overall prevalence of pouch failure is 6%. Recent meta-analyses by Heuthorst et al. [34] and Emile et al. [35] report pouch failure rates of 7.7% and 7.5%, respectively. However, these studies also assessed patients with intermediate colitis, cancer and Crohn’s disease along with UC which may have a compounding detrimental effect on pouch outcomes. Prior studies have shown that complications predominantly occur within the first few months of surgery [36]. This suggested that the IPAA may withstand the test of time with a relatively stable failure rate. This is echoed by Lorenzo et al. [11], who found that despite slightly worsening function over a period of 20 years, failure rates remained stable while quality of life measurements remained high. However, there are relatively few studies that provide long follow-up periods, with only 7 articles in our meta-analysis following patient for ≥ 10 years. This would have added another dimension to our understanding of pouch failure, particularly with there being a suggestion of increasing failure rates with time [36, 37]. Despite this, our analysis did not show any evidence of publication bias.

Our results show that the year of publication did not affect pouch failure rates. This is despite several advancements to the procedure in recent years such as the advent different pouch designs and the introduction of the laparoscopic and robotic approaches [38, 39]. Although not specifically investigated in our study, there is evidence to suggest that these advancements have not only led to faster recovery times and progression to restoration of intestinal continuity, but have also led to better functional outcomes over time which have been reported to have a positive impact on patients’ quality of life [3, 38, 40, 41]. This may prove to be useful when counselling patients on the risks and benefits of the procedure.

Additionally, the analysis demonstrated significant heterogeneity overall, and in each follow-up period. This could reflect the differences in study design, follow-up periods, baseline patient characteristics, type of IPAA performed and type of centre the procedure was performed in. It is also worth considering the differences in practice between countries and how this may impact the management of UC as a whole.

Several causes of pouch failure were cited in the literature such as Crohn’s disease, pouchitis, fistulae, pelvic sepsis and leaks. Additionally, several risk factors for pouch failure have been identified such as male gender, high BMI, advanced age and extraintestinal manifestation of UC such as erythema nodosum [42]. However, only a few studies reported the exact figures, making it difficult to ascertain the correlation between the complications and pouch failure. Heuthorst et al. [34] showed that the pouch failure was significantly correlated with fistulae and pelvic sepsis. Of note, the study included patients with Crohn’s disease, indeterminate colitis, familial adenomatous polyposis, and colorectal cancer along with UC. Nonetheless, it may be used to guide future follow-up. The aim of this study was to provide an estimate of the prevalence of pouch failure in patients with UC. This may guide future studies and power calculations. We excluded studies where pouches were originally constructed for CD as the literature suggests that they have a higher incidence of failure and was deemed to be a relative contraindication [43] Additionally, significant differences exist in the definition and terminology used when discussing CD of the pouch which may result in its overdiagnosis [44]. Therefore, including these studies may have skewed our results. We also excluded studies assessing patients with primary sclerosing cholangitis as it is known to have a significant impact on pouch failure and would also skew the results [45]. The lack of a definition has resulted in large discrepancies in the reported incidence of pouch failure secondary to CD and may benefit from a metanalysis of its own.

This is the first meta-analysis evaluating the literature from the inception of IPAA to May 2021. The large sample size of 23,389 patients allowed us to give a comprehensive estimate of the prevalence of pouch failure in UC. Our bubble plot shows that the year of publication did not have a significant effect on pouch failure rates, allowing us to include all eligible studies from 1978 to 2021. Furthermore, we assessed how failure rates change over time which would assist patients in making informed decisions. Additionally, we excluded studies with small sample sizes to minimise the risk of selection bias.

Limitations of this study should also be noted. Firstly, the studies included are observational in nature with no prospective randomised studies being available. This, however, is unavoidable in view of these being the mainstay of available evidence in this area. Secondly, most studies included were performed on western populations which may impact the article’s generalisability. As our aims were to assess overall pouch failure, we examined studies looking at whole UC populations and excluded articles looking at specific surgical techniques. Additionally, we attempted to evaluate the prevalence of pouch failure at multiple time points to provide granularity. Unfortunately, most of the studies that met our inclusion criteria did not report failure at multiple time points and yielded insufficient data to allow for meaningful analysis. Several articles cited CD as a cause of pouch failure despite operating on patients with UC exclusively. Additionally, fistulae may have occurred secondary to CD as well as surgical complications and ischaemia. In an ideal situation, we would have reanalysed the data, excluding patients with a postoperative diagnosis of CD. Unfortunately, the data lacked granularity and proved to be insufficient when undertaking any meaningful analyses.

Future studies should aim to report the causes of pouch failure using established definitions at various timepoints. This would allow us to find optimal follow-up periods, monitoring modalities and management plans in order to minimise the risk of pouch failure.

## Conclusion

This systematic review and meta-analysis of patients with UC who underwent IPAA demonstrates an overall prevalence of pouch failure of 6%. Despite the intricacies of the subject matter, the question of pouch failure is an important one to address, with these data being particularly important for counselling patients considering the procedure. Importantly, for those patients with UC being considered for a pouch, their disease course has often resulted in both physical and psychological morbidity and hence providing accurate expectations is vital.

## Supplementary information

Below is the link to the electronic supplementary material.

Supplementary file1 (DOCX 13 KB)

Supplementary file2 (XLSX 16 KB)

Supplementary file3 (DOCX 141 KB)

## Data Availability

The data underlying this article are available in the article and in its online supplementary material.

## Code availability

Available on request by emailing corresponding author.
