# Supplementary material for: Prevalence of ‘pouch failure’ of the ileoanal pouch in ulcerative colitis: a systematic review and meta-analysis
Source: Int J Colorectal Dis. 2021 Nov 26;37(2):357–64. doi: 10.1007/s00384-021-04067-6 (PMC8803821; doi:10.1007/s00384-021-04067-6)
Supplement: Supplementary file 1 — Supplementary file1 (DOCX 13 KB) [file 384_2021_4067_MOESM1_ESM.docx]

**Supplementary Table 1: Search terms/ strategy**

| Search Terms/ strategy |
| --- |
| 1 Pouch*.af. 60371  2 Ileoanal*.af. 4159  3 IPAA.af. 3538  4 Proctocolectomy, Restorative.af. 3450  5 Colonic Pouches.af. 1743  6 Colitis, Ulcerative.af. 37353  7 UC.af. 146602  8 ileal pouch anal anastomosis.af. 5795  9 ileoanal pouch.af. 1087  10 ileal pouch anal anastomosis.af. 5795  11 Redo-pouch.af. 73  12 Reoperation.af. 211475  13 Ileostomy.af. 28548  14 stoma.af. 35949  15 Surgical Stomas.af. 2102  16 failure.af. 2610375  17 diversion.af. 60524  18 1 or 2 or 3 or 4 or 5 or 8 or 9 62521  19 6 or 7 174578  20 11 or 12 or 13 or 14 or 15 or 16 or 17 2872532  21 18 and 19 and 20 2247  22 remove duplicates from 21 1917 |
