# Supplementary material for: Prevalence of ‘pouch failure’ of the ileoanal pouch in ulcerative colitis: a systematic review and meta-analysis
Source: Int J Colorectal Dis. 2021 Nov 26;37(2):357–64. doi: 10.1007/s00384-021-04067-6 (PMC8803821; doi:10.1007/s00384-021-04067-6)

**Supplementary Figure 1:** Funnel plot of pouch failure with follow up < 5 years (Eggers test = 0.1263506).


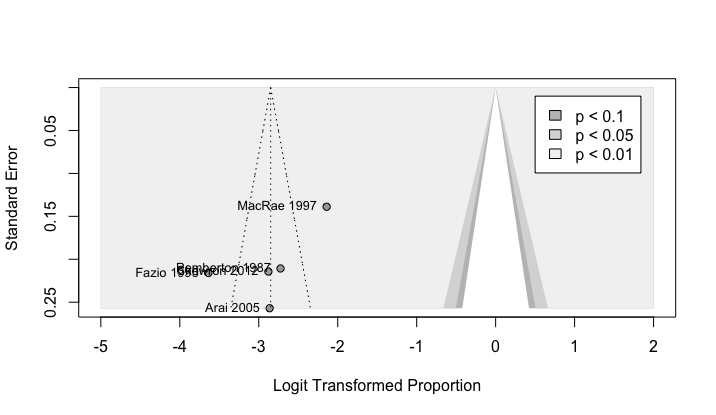


**Supplementary Figure 2:** Funnel plot of pouch failure with follow up ≥5 but <10 years (Eggers test = 0.9885051)


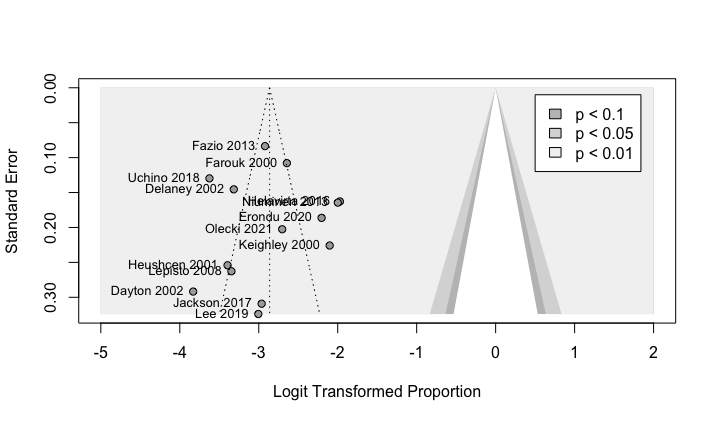


**Supplementary Figure 3:** Funnel plot of pouch failure with follow up ≥ 10 (Eggers test not possible due to insufficient data)


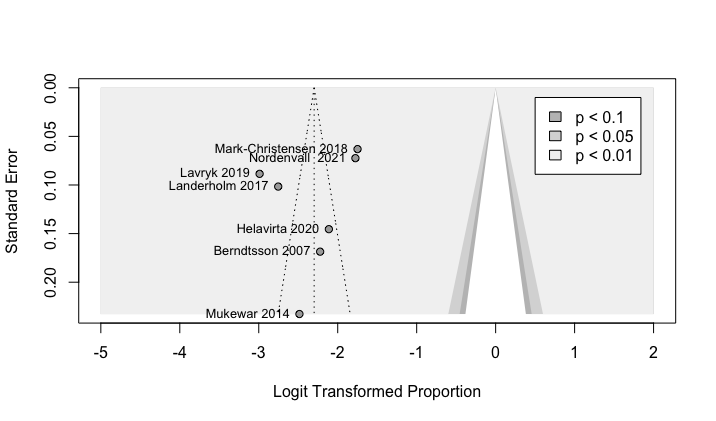

Supplement: Supplementary file 3 — Supplementary file3 (DOCX 141 KB) [file 384_2021_4067_MOESM3_ESM.docx]
